# Alcohol Dependence, Withdrawal, and Relapse

**Published:** 2008

**Authors:** Howard C. Becker

**Keywords:** Alcoholism, alcohol dependence, alcohol and other drug (AOD) effects and consequences, neuroadaptation, AOD withdrawal syndrome, AOD dependence relapse, pharmacotherapy, human studies, animal studies

## Abstract

Continued excessive alcohol consumption can lead to the development of dependence that is associated with a withdrawal syndrome when alcohol consumption is ceased or substantially reduced. This syndrome comprises physical signs as well as psychological symptoms that contribute to distress and psychological discomfort. For some people the fear of withdrawal symptoms may help perpetuate alcohol abuse; moreover, the presence of withdrawal symptoms may contribute to relapse after periods of abstinence. Withdrawal and relapse have been studied in both humans and animal models of alcoholism. Clinical studies demonstrated that alcohol-dependent people are more sensitive to relapse-provoking cues and stimuli than nondependent people, and similar observations have been made in animal models of alcohol dependence, withdrawal, and relapse. One factor contributing to relapse is withdrawal-related anxiety, which likely reflects adaptive changes in the brain in response to continued alcohol exposure. These changes affect, for example, the body’s stress response system. The relationship between withdrawal, stress, and relapse also has implications for the treatment of alcoholic patients. Interestingly, animals with a history of alcohol dependence are more sensitive to certain medications that impact relapse-like behavior than animals without such a history, suggesting that it may be possible to develop medications that specifically target excessive, uncontrollable alcohol consumption.

The development of alcohol dependence is a complex and dynamic process. Many neurobiological and environmental factors influence motivation to drink (Grant 1995; Samson and Hodge 1996; Vengeliene et al. 2008; Weiss 2005). At any given time, an individual’s propensity to imbibe is thought to reflect a balance between alcohol’s positive reinforcing (i.e., rewarding) effects, such as euphoria and reduction of anxiety (i.e., anxiolysis), and the drug’s aversive effects, which typically are associated with negative consequences of alcohol consumption (e.g., hangover or withdrawal symptoms). Memories associated with these rewarding and aversive qualities of alcohol, as well as learned associations between these internal states and related environmental stimuli or contexts, influence both the initiation and regulation of intake. These experiential factors, together with biological and environmental influences and social forces, are central to the formation of expectations about the consequences of alcohol use. These expectations, in turn, shape an individual’s decision about engaging in drinking behavior.

The nature of and extent to which these factors are operable in influencing decisions about drinking not only vary from one individual to another but also depend on the stage of addiction—that is, whether the drinker is at the stage of initial experience with alcohol, early problem drinking, or later excessive consumption associated with dependence. Although many people abuse alcohol without meeting the criteria for alcohol dependence,^1^ continued excessive alcohol consumption can lead to the development of dependence. Neuroadaptive changes that result from continued alcohol use and abuse (which manifest as tolerance and physiological dependence) are thought to be crucial in the transition from controlled alcohol use to more frequent and excessive, uncontrollable drinking (Koob and Le Moal 2008). Indeed, for some dependent individuals, the fear that withdrawal symptoms might emerge if they attempt to stop or significantly curtail drinking may prominently contribute to the perpetuation of alcohol use and abuse.

This article will provide an overview of the basic features of alcohol dependence and the associated withdrawal syndrome, emphasizing those components of withdrawal that especially are thought to contribute to the problem of relapse. It will present evidence from both clinical and experimental studies that highlights long-lasting physiological and emotional changes which are characteristic of dependence and have been postulated to play a key role in persistent vulnerability to relapse. In particular, it will review animal models of alcohol dependence and withdrawal, as well as models of self-administration, that have helped researchers elucidate brain mechanisms underlying relapse and excessive drinking associated with dependence.

## Alcohol Withdrawal

When an alcohol-dependent individual abruptly terminates or substantially reduces his or her alcohol consumption, a characteristic withdrawal syndrome ensues. In general, alcohol acts to suppress central nervous system (CNS) activity, and, as with other CNS depressants, withdrawal symptoms associated with cessation of chronic alcohol use are opposite in nature to the effects of intoxication. Typical clinical features of alcohol withdrawal include the following (Becker 2000; Hall and Zador 1997; Saitz 1998):

- Signs of heightened autonomic nervous system^2^ activation, such as rapid heartbeat (i.e., tachycardia), elevated blood pressure, excessive sweating (i.e., diaphoresis), and shaking (i.e., tremor);
- Excessive activity of the CNS (i.e., CNS hyperexcitability) that may culminate in motor seizures; and
- Hallucinations and delirium tremens in the most severe form of withdrawal.

In addition to physical signs of withdrawal, a constellation of symptoms contributing to a state of distress and psychological discomfort constitute a significant component of the withdrawal syndrome (Anton and Becker 1995; Roelofs 1985; Schuckit et al. 1998). These symptoms include emotional changes such as irritability, agitation, anxiety, and dysphoria, as well as sleep disturbances, a sense of inability to experience pleasure (i.e., anhedonia), and frequent complaints about “achiness,” which possibly may reflect a reduced threshold for pain sensitivity. Many of these signs and symptoms, including those that reflect a negative-affect state (e.g., anxiety, distress, and anhedonia) also have been demonstrated in animal studies involving various models of dependence (Becker 2000).

Although many physical signs and symptoms of withdrawal typically abate within a few days, symptoms associated with psychological distress and dysphoria may linger for protracted periods of time (Anton and Becker 1995; De Soto et al. 1985; Martinotti et al. 2008). The persistence of these symptoms (e.g., anxiety, negative affect, altered reward set point manifesting as dysphoria and/or anhedonia) may constitute a significant motivational factor that leads to relapse to heavy drinking.

## Studying Alcohol Relapse Behavior

Relapse may be defined as the resumption of alcohol drinking following a prolonged period of abstinence. Clinically, vulnerability to relapse commonly is associated with an intense craving or desire to drink. Although a precise definition for craving remains elusive (Anton 1999; Koob 2000; Littleton 2000), and there even is some debate about the role of craving in relapse (Miller and Gold 1994; Rohsenow and Monti 1999; Tiffany and Carter 1998), there is no question that relapse represents a prevalent and significant problem in alcoholism. In fact, given the high rate of recidivism in alcoholism, relapse clearly is a major impediment to treatment efforts. Consequently, substantial research efforts have been directed at modeling relapse behavior, as well as elucidating neural substrates and environmental circumstances that are associated with or promote excessive drinking.

Events that potently trigger relapse drinking fall into three general categories: exposure to small amounts of alcohol (i.e., alcohol-induced priming), exposure to alcohol-related (i.e., conditioned) cues or environmental contexts, and stress. Clinical laboratory studies have found that compared with control subjects, alcohol-dependent people are more sensitive to the ability of these stimuli and events to elicit craving and negative affect, which in turn presumably drives an increased desire to drink (Fox et al. 2007; Sinha et al. 2008). The combination of these clinical laboratory procedures with neuroimaging techniques has proven to be a powerful tool allowing investigators to identify brain regions that are more strongly activated in alcohol-dependent subjects than in control subjects when they are exposed to these stimuli/events (George et al. 2001; Myrick et al. 2004; Wrase et al. 2002). Similar experimental procedures have been employed to evaluate the ability of pharmacotherapeutics to quell craving and temper the brain activation provoked by alcohol-related cues in humans (Anton et al. 2004; George et al. 2008; Myrick et al. 2007, 2008; O’Malley et al. 2002).

More detailed insight regarding mechanisms underlying fundamental changes in brain function that occur as a consequence of dependence and which relate to enduring relapse vulnerability have been gained through research in animals. Several animal models have been used to study alcohol self-administration behavior and the issue of relapse (for reviews, see Le and Shaham 2002; Sanchis-Segura and Spanagel 2006; Weiss 2005). In one type of model, animals with a long history of daily access to alcohol are abruptly denied access to the drug. When alcohol is reintroduced after this period of “forced” (i.e., experimenter-induced) abstinence, the animals exhibit a transient increase in alcohol consumption. This alcohol deprivation effect has been demonstrated using both measures of voluntary alcohol consumption and operant procedures^3^ (Heyser et al. 1997; Sinclair 1979; Spanagel and Holter 1999). Another model frequently used to study alcohol (and other drug) relapse behavior involves operant reinstatement procedures (Shaham et al. 2003). In this model, animals first are trained to respond for access to alcohol (i.e., to receive the reinforcement provided by alcohol). Then, the response-contingent reinforcement is interrupted with extinction training—that is, even if the animals perform the required response, they do not receive alcohol; as a result, the animals eventually reduce or even completely stop responding. When the animals then are exposed again to small alcohol doses, environmental stressors, or stimuli previously associated with delivery of alcohol (i.e., conditioned cues), they resume responding (to varying degrees)—as if “seeking” alcohol reinforcement (Le et al. 1998, 2000; Weiss et al. 2001). This renewed alcohol-seeking behavior becomes even more robust when several of these relevant stimuli are presented in combination (Backstrom and Hyytia 2004; Liu and Weiss 2002*b*). Interestingly, this reinstatement of alcohol responding occurs even though the animals still do not receive alcohol reinforcement.

This experimental design can be further modified by the use of discriminative contextual cues. This means that certain contextual cues (e.g., a unique odor or testing environment) will indicate to the animal that responding will pay off with delivery of alcohol reinforcement, whereas a different contextual cue is used to signal that responding will not result in access to alcohol. If the responding is extinguished in these animals (i.e., they cease to respond because they receive neither the alcohol-related cues nor alcohol), presentation of a discriminative cue that previously signaled alcohol availability will reinstate alcohol-seeking behavior. This renewed alcohol-seeking behavior can be observed even after a long period of time has elapsed since the animals last were given an opportunity to self-administer alcohol, suggesting that these contextual cues can serve as powerful triggers for relapse-like behavior (Ciccocioppo et al. 2001; Katner and Weiss 1999; Katner et al. 1999). Additional studies (Chaudhri et al. 2008; Zironi et al. 2006) found that reexposure of the animals to the general environmental context in which they could self-administer alcohol not only enhanced subsequent alcohol responding but also modulated the ability of alcohol-conditioned cues to reinstate alcohol-seeking behavior.

Finally, and perhaps most importantly, animals used in all of these models generally have demonstrated sensitivity to treatment with various medications that have been shown to be clinically effective in preventing and/or retarding alcohol relapse (Burattini et al. 2006; Heilig and Egli 2006; Le and Shaham 2002; Marinelli et al. 2007*b*; Spanagel and Kiefer 2008). From a clinical standpoint, this is important because it underscores the value of these models in identifying and evaluating new treatment strategies that may be more effective in battling the problem of relapse.

## Alcohol Dependence, Withdrawal, and Relapse

**Figure 1:**
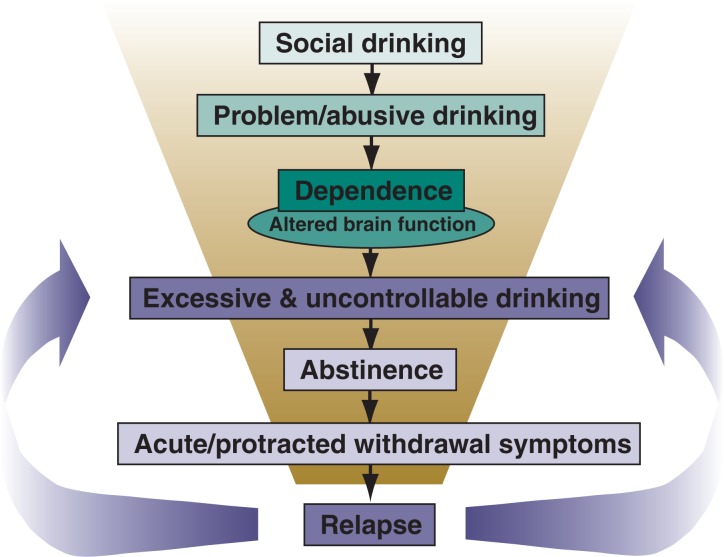
Schematic illustration of how problem drinking can lead to the development of dependence, repeated withdrawal experiences, and enhanced vulnerability to relapse. Alcohol dependence is characterized by fundamental changes in the brain’s reward and stress systems that manifest as withdrawal symptoms when alcohol consumption is stopped or substantially reduced. These changes also are purported to fuel motivation to reengage in excessive drinking behavior. Repeated bouts of heavy drinking interspersed with attempts at abstinence (i.e., withdrawal) may result in sensitization of withdrawal symptoms, especially symptoms that contribute to a negative emotional state. This, in turn, can lead to enhanced vulnerability to relapse as well as favor perpetuation of excessive drinking.

As mentioned earlier, alcohol addiction is a complex and dynamic process (see figure 1). Prolonged excessive alcohol consumption sets in motion a host of neuroadaptive changes in the brain’s reward and stress systems (for reviews, see Hansson et al. 2008; Heilig and Koob 2007; Koob and Le Moal 2008; Vengeliene et al. 2008). The development of alcohol dependence is thought to reflect an allostatic state—that is, a state in which the chronic presence of alcohol produces a constant challenge to regulatory systems that attempt (but ultimately fail) to defend the normal equilibrium of various internal processes (i.e., homeostatic set points). In the dependent individual, this allostatic state is fueled by progressive dysregulation of the brain’s reward and stress systems beyond their normal homeostatic limits (Koob 2003; Koob and Le Moal 2001). These neuroadaptive changes associated with dependence and withdrawal are postulated to impact the rewarding effects of alcohol and, consequently, contribute to the transition from controlled alcohol use to more excessive, uncontrollable drinking. Manifestations of these perturbations in brain reward and stress systems also appear to mediate the myriad symptoms of alcohol withdrawal, as well as underlie persistent vulnerability to relapse.

As noted above, clinical laboratory studies have shown that alcohol-dependent people are more sensitive to relapse-provoking cues/stimuli compared with control subjects. By definition, alcohol-dependent subjects also are heavier drinkers and (too) often experience an insidious return to excessive levels of alcohol consumption once a “slip” occurs after abstinence. Not surprisingly, numerous rodent and primate models have been employed to examine the influence of dependence on relapse. Early studies using these animal models generally yielded equivocal findings, most likely because investigators used procedures that neither sufficiently established alcohol’s positive reinforcing effects prior to dependence induction nor optimized the development of alcohol’s negative reinforcing capacity (i.e., the animals did not have an opportunity to associate alcohol drinking with alleviation of withdrawal symptoms) (Meisch 1983; Meisch and Stewart 1994).

More recent studies that have incorporated these procedural considerations, however, have demonstrated increased alcohol responding and/or drinking in dependent compared with nondependent mice (Becker and Lopez 2004; Chu et al. 2007; Dhaher et al. 2008; Finn et al. 2007; Lopez and Becker 2005) and rats (O’Dell et al. 2004; Rimondini et al. 2003; Roberts et al. 2000; Sommer et al. 2008; Valdez et al. 2002). Moreover, in some studies, the enhanced alcohol consumption in dependent animals during withdrawal produced blood and brain alcohol levels that nearly reached levels attained during the initial chronic alcohol exposure which had produced the dependent state (Griffin et al. 2008; Roberts et al. 2000). Also, consistent with the findings of clinical studies, animals with a history of alcohol dependence exhibited exaggerated sensitivity to alcohol-related cues and various stressors that lead to enhanced alcohol-seeking behavior (Gehlert et al. 2007; Liu and Weiss 2002*b*; Sommer et al. 2008). In many instances, these effects were observed long after the animals had experienced chronic alcohol exposure (Lopez and Becker 2005; Rimondini et al. 2003; Valdez et al. 2002). Finally, experience with repeated cycles of chronic alcohol exposure and withdrawal not only led to an exacerbation of the physiological symptoms of withdrawal but also to enhanced susceptibility to relapse (for more information on this issue, see the sidebar, “Repeated Alcohol Withdrawals: Sensitization and Implications for Relapse”). Thus, a growing body of evidence indicates that alcohol dependence and withdrawal experiences significantly contribute to enhanced relapse vulnerability as well as favor sustained high levels of alcohol drinking once a “slip” occurs.

## Role of Withdrawal-Related Stress and Anxiety in Relapse

As previously noted, increased anxiety represents a significant component of the alcohol withdrawal syndrome. Importantly, this negative-affect state may contribute to increased risk for relapse as well as perpetuate continued use and abuse of alcohol (Becker 1999; Driessen et al. 2001; Koob 2003; Roelofs 1985). Indeed, both preclinical and clinical studies suggest a link between anxiety and propensity to self-administer alcohol (Henniger et al. 2002; Spanagel et al. 1995; Willinger et al. 2002).

Various experimental procedures have been used to demonstrate increased behavioral anxiety in animal models of alcohol dependence and withdrawal (Becker 2000; Kliethermes 2005). Many of these models involve procedures that exploit the natural tendency of rodents to avoid environments (e.g., bright open spaces) that may be considered dangerous or threatening, thereby eliciting an internal state of fear or anxiety. Other models assess an animal’s propensity to engage in social interaction with another (unfamiliar) animal of the same species (Overstreet et al. 2002) or response under conflict situations (Sommer et al. 2008). Finally, some models use operant discrimination procedures to train animals to discern subjective (i.e., interoceptive) cues associated with an anxiety-inducing (i.e., anxiogenic) state experienced during withdrawal (Gauvin et al. 1992; Lal et al. 1988).

Alcohol withdrawal–related anxiety is thought to reflect manifestations of numerous adaptive changes in the brain resulting from prolonged alcohol exposure, most notably alterations in the stress systems active in the brain and the body’s hormone (i.e., endocrine) circuits. The hormonal stress response is mediated by a system known as the hypothalamic–pituitary–adrenocortical (HPA) axis. Within this system, stress induces the release of the hormone corticotrophin-releasing factor (CRF) from a brain area called the hypothalamus. CRF acts on the pituitary gland located directly below the hypothalamus, where it initiates the production of a molecule called proopiomelanocortin (POMC). This compound is processed further into smaller molecules, such as β-endorphin and adrenocorticotropic hormone (ACTH). ACTH is carried via the blood stream to the adrenal glands (which are located atop the kidneys), where it induces the release of stress hormones (i.e., glucocorticoids) that then act on target cells and tissues throughout the body (including the brain). The main glucocorticoid in humans and other primates is cortisol; the main glucocorticoid in rodents is corticosterone.

**Repeated Alcohol Withdrawals**

**Sensitization and Implications for Relapse**

Given that alcoholism is a chronic relapsing disease, many alcohol-dependent people invariably experience multiple bouts of heavy drinking interspersed with periods of abstinence (i.e., withdrawal) of varying duration. A convergent body of preclinical and clinical evidence has demonstrated that a history of multiple detoxification/withdrawal experiences can result in increased sensitivity to the withdrawal syndrome—a process known as “kindling” (Becker and Littleton 1996; Becker 1998). For example, clinical studies have indicated that a history of multiple detoxifications increases a person’s susceptibility to more severe and medically complicated withdrawals in the future (e.g., Booth and Blow 1993). Similarly, animal studies have demonstrated sensitization of electrographic and behavioral measures of withdrawal seizure activity in mice following multiple withdrawals compared with animals tested after a single withdrawal episode, even if both groups of animals had been exposed to the same total amount of alcohol (e.g., Becker and Hale 1993; Becker 1994; Veatch and Becker 2002, 2005).

**Effects of Repeated Withdrawals on Emotional State and Stress Response**

Most studies demonstrating this sensitization or “kindling” of alcohol withdrawal primarily have focused on withdrawal-related excessive activity (i.e., hyperexcitability) of the central nervous system (CNS), as indicated by seizure activity, because this parameter is relatively easy to observe in experimental as well as clinical settings. More recently, however, researchers have been turning their attention to the evaluation of changes in withdrawal symptoms that extend beyond physical signs of withdrawal—that is, to those symptoms that fall within the domain of psychological distress and dysphoria. This new focus is clinically relevant because these symptoms (e.g., anxiety, negative affect, and altered reward set point) may serve as potent instigators driving motivation to drink (Koob and Le Moal 2008). Sensitization resulting from repeated withdrawal cycles and leading to both more severe and more persistent symptoms therefore may constitute a significant motivational factor that underlies increased risk for relapse (Becker 1998, 1999).

Furthermore, multiple withdrawal episodes provide repeated opportunities for alcohol-dependent individuals to experience the negative reinforcing properties of alcohol—that is, to associate alcohol consumption with the amelioration of the negative consequences (e.g., withdrawal-related malaise) experienced during attempts at abstinence. This association not only may serve as a powerful motivational force that increases relapse vulnerability, but also favors escalation of alcohol drinking and sustained levels of potentially harmful drinking. Thus, for many dependent individuals, repeated withdrawal experiences may be especially relevant in shaping motivation to seek alcohol and engage in excessive drinking behavior.

Support for the notion that repeated withdrawal experience progressively intensifies withdrawal symptoms—which, in turn, impacts relapse vulnerability and facilitates transition to uncontrollable drinking— primarily has come from studies involving animal models. For example, animals with a history of chronic alcohol exposure and repeated withdrawal experiences were shown to exhibit enhanced withdrawal-related anxiety, as measured in a variety of behavioral tasks (Overstreet et al. 2002, 2004; Sommer et al. 2008; Zhang et al. 2007). Moreover, such a history enhanced the animals’ sensitivity to various stressors, as measured by the stressors’ ability to activate the body’s stress response system (i.e., the hypothalamic–pituitary–adrenocortical [HPA] axis) (Becker 1999), to produce anxiety-like behavior (Breese et al. 2005; Sommer et al. 2008), and to trigger relapse-like behavior (Ciccocioppo et al. 2003). In all these cases, increased activity of a signaling molecule called corticotropin-releasing factor (CRF) was found to be a critical mediating factor. This finding lends support to the idea that enhanced CRF activity represents a key neuroadaptive change that is fueled by repeated withdrawal experience and which drives (at least in part) the motivation to drink as well as amplifies responsiveness to stimuli/events that provoke relapse (Heilig and Koob 2007; Koob and Le Moal 2008). (For more information on the body’s stress response, including the HPA axis and CRF, see the main article.)

Additional evidence indicates that behavioral measures indicating a reduced sensitivity to rewarding stimuli (i.e., anhedonia) are exaggerated in rats that experience withdrawal from repeated alcohol injections compared with rats tested during withdrawal from a single alcohol injection (Schulteis and Liu 2006). Finally, a history of multiple withdrawal experiences can exacerbate cognitive deficits and disruption of sleep during withdrawal (Borlikova et al. 2006; Stephens et al. 2005; Veatch 2006). Taken together, these results indicate that chronic alcohol exposure involving repeated withdrawal experiences exacerbates withdrawal symptoms that significantly contribute to a negative emotional state, which consequently renders dependent subjects more vulnerable to relapse.

**Effects of Repeated Withdrawals on Tolerance to Subjective Alcohol Effects and Alcohol Self-Administration**

Researchers also have explored the effects of repeated withdrawal episodes on the perceived subjective effects of alcohol. In animal studies using operant discrimination procedures,^1^ the animals’ ability to detect (perceive) the subjective cues associated with alcohol intoxication was diminished during withdrawal from chronic alcohol exposure, and this tolerance effect was enhanced in mice that experienced multiple withdrawals during the course of the chronic alcohol treatment (Becker and Baros 2006). Similarly, rats with a history of repeated cycles of chronic alcohol exposure and withdrawal exhibited long-lasting tolerance to the sedative/ hypnotic effects of alcohol (Rimondini et al. 2008). Because changes in sensitivity as well as in the ability to detect (perceive) subjective effects associated with alcohol intoxication may influence decisions about drinking and, in particular, control over the amount consumed during a given drinking occasion, these observations may be relevant to the problem of relapse and excessive drinking. Indeed, clinical studies have indicated that heavy drinkers exhibit a reduced capacity to detect (discriminate) internal cues associated with alcohol intoxication (Hiltunen 1997; Jackson et al. 2001; Schuckit and Klein 1991). Future studies will need to further explore the potential relationship between increased tolerance to subjective effects of alcohol produced by repeated withdrawal experience and enhanced propensity to imbibe.

More direct evidence supporting increased alcohol consumption as a consequence of repeated withdrawal experience comes from animal studies linking dependence models with self-administration procedures. For example, rats exposed to chronic alcohol treatment interspersed with repeated withdrawal episodes consumed significantly more alcohol than control animals under free-choice, unlimited access conditions (Rimondini et al. 2002, 2003; Sommer et al. 2008). Similar results have been reported in mice, with voluntary alcohol consumption assessed using a limited access schedule (Becker and Lopez 2004; Dhaher et al. 2008; Finn et al. 2007; Lopez and Becker 2005). Likewise, studies using operant procedures have demonstrated increased alcohol self-administration in mice (Chu et al. 2007; Lopez et al. 2008) and rats (O’Dell et al. 2004; Roberts et al. 1996, 2000) with a history of repeated chronic alcohol exposure and withdrawal experience. Further, the amount of work mice (Lopez et al. 2008) and rats (Brown et al. 1998) were willing to expend in order to receive alcohol reinforcement was significantly increased following repeated withdrawal experience. This suggests that the reinforcing value of alcohol may be enhanced as a result of experiencing repeated opportunities to respond for access to alcohol in the context of withdrawal.

Enhanced alcohol responding/ intake in dependent animals occurred well beyond the period of acute withdrawal, and escalation of alcohol self-administration was especially facilitated when dependence was induced by delivering chronic alcohol in an intermittent rather than continuous fashion (Lopez and Becker 2005; O’Dell et al. 2004). This latter finding suggests that elevated alcohol self-administration does not merely result from long-term alcohol exposure per se, but rather that repeated withdrawal experiences underlie enhanced motivation for alcohol seeking/consumption. Additionally, the more cycles of chronic alcohol exposure and withdrawal the animals were exposed to, the more alcohol they ingested and the longer (i.e., for several weeks) the enhanced alcohol intake was sustained following the final withdrawal episode compared with a separate group of nondependent mice (Lopez and Becker 2005). This effect apparently was specific to alcohol because repeated chronic alcohol exposure and withdrawal experience did not produce alterations in the animals’ consumption of a sugar solution (Becker and Lopez 2004). More detailed analyses of the pattern of alcohol consumption revealed that dependent mice not only consumed more alcohol than nondependent animals over the entire 2-hour period during which they had access to alcohol, but that the rate of consumption was faster and progressively increased with successive withdrawal test periods (Griffin et al. 2008).

**Figure 2:**
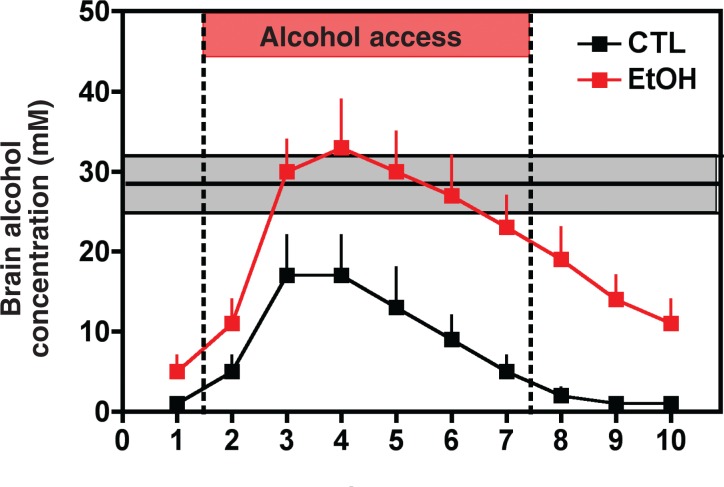
Enhanced voluntary alcohol drinking in dependent mice produced brain alcohol concentrations similar to those achieved during the chronic alcohol exposure that initially rendered the animals dependent. Samples were collected from the nucleus accumbens of alcohol-dependent mice that had undergone three cycles of chronic intermittent alcohol vapor exposure (red symbols) and nondependent controls (black symbols). Samples were taken before, during, and after the 2-hour drinking session, when the mice had the opportunity to voluntarily drink alcohol (15 percent vol/vol) or water. Alcohol intake during the drinking session was 3.04 ± 0.15 g/kg for dependent mice and 2.32 ± 0.28 g/kg for nondependent mice. The red bar indicates the 2-hour drinking session. Horizontal lines and shaded area represent brain alcohol levels (means ± SEM) measured in the dependent mice during chronic intermittent alcohol exposure (28.4 ± 3.5 mM). NOTE: Brain alcohol concentrations (mM) were measured in microdialysis samples collected from the nucleus accumbens. Values are corrected for calculated recovery rates (∼10 percent) for microdialysis probes. SEM = standard error of the mean. SOURCE: Data are adapted from Griffin et al. 2008.

In both mice and rats, enhanced alcohol self-administration following repeated cycles of withdrawal was associated with significantly higher resultant blood alcohol levels compared with the levels achieved by nondependent animals (Becker and Lopez 2004; Roberts et al. 2000). The greater (and faster) alcohol intake exhibited by dependent mice also lead to significantly higher peak and more sustained alcohol concentrations in the brain compared with the levels achieved after alcohol consumption in nondependent animals (Griffin et al. 2008). Finally, greater voluntary alcohol consumption in dependent mice produced brain alcohol concentrations that approximated those levels experienced during the chronic intermittent alcohol exposure which had rendered the animals dependent in the first place (see figure 2, main section). Although it is tempting to speculate that dependent animals increase voluntary alcohol drinking to attain blood and brain alcohol levels in a range consistent with sustaining dependence, the extent to which resultant brain alcohol concentrations help drive as well as perpetuate enhanced alcohol drinking in dependent animals remains to be determined.

**Effects of Repeated Withdrawals on Sensitivity to Treatment**

Some studies using animal models involving repeated withdrawals have demonstrated altered sensitivity to treatment with medications designed to quell sensitized withdrawal symptoms (Becker and Veatch 2002; Knapp et al. 2007; Overstreet et al. 2007; Sommer et al. 2008; Veatch and Becker 2005). Moreover, after receiving some of these medications, animals exhibited lower relapse vulnerability and/or a reduced amount consumed once drinking was (re)-initiated (Ciccocioppo et al. 2003; Finn et al. 2007; Funk et al. 2007; Walker and Koob 2008). These findings have clear clinical relevance from a treatment perspective. Indeed, clinical investigations similarly have reported that a history of multiple detoxifications can impact responsiveness to and efficacy of various pharmacotherapeutics used to manage alcohol dependence (Malcolm et al. 2000, 2002, 2007). Future studies should focus on elucidating neural mechanisms underlying sensitization of symptoms that contribute to a negative emotional state resulting from repeated withdrawal experience. Such studies will undoubtedly reveal important insights that spark development of new and more effective treatment strategies for relapse prevention as well as aid people in controlling alcohol consumption that too often spirals out of control to excessive levels.

—*Howard Becker, Ph.D.*

1In operant procedures, animals must first perform a certain response (e.g., press a lever) before they receive a stimulus (e.g., a small amount of alcohol). By modifying the required response (e.g., increasing the number of lever presses required before the alcohol is delivered) researchers can determine the motivational value of the stimulus for the animal.

ReferencesAmerican Psychiatric AssociationDiagnostic and Statistical Manual of Mental Disorders4th EditionArlington, VAAmerican Psychiatric Association1994BeckerHCPositive relationship between the number of prior ethanol withdrawal episodes and the severity of subsequent withdrawal seizuresPsychopharmacology11626321994786292710.1007/BF02244867BeckerHCKindling in alcohol withdrawalAlcohol Health & Research World222533199815706729PMC6761822BeckerHCAlcohol withdrawal: Neuroadaptation and sensitizationCNS Spectrums438651999BeckerHCBarosAMEffect of duration and pattern of chronic ethanol exposure on tolerance to the discriminative stimulus effects of ethanol in C57BL/6J miceJournal of Pharmacology and Experimental Therapeutics31987187820061691456010.1124/jpet.106.108795BeckerHCHaleRLRepeated episodes of ethanol withdrawal potentiate the severity of subsequent withdrawal seizures: An animal model of alcohol withdrawal “kindling”Alcoholism: Clinical and Experimental Research17194981993845221210.1111/j.1530-0277.1993.tb00731.xBeckerHCLittletonJMThe alcohol withdrawal “kindling” phenomenon: Clinical and experimental findingsAlcoholism: Clinical and Experimental Research20121A124A199610.1111/j.1530-0277.1996.tb01760.x8947249BeckerHCLopezMFIncreased ethanol drinking after repeated chronic ethanol exposure and withdrawal experience in C57BL/6 miceAlcoholism: Clinical and Experimental Research281829183820041560859910.1097/01.alc.0000149977.95306.3aBeckerHCVeatchLMEffects of lorazepam treatment for multiple ethanol withdrawals in miceAlcoholism: Clinical and Experimental Research26371380200211923591BoothBMBlowFCThe kindling hypothesis: Further evidence from a U.S. national study of alcoholic menAlcohol & Alcoholism2859359819938274184BorlikovaGGElbersNAStephensDNRepeated withdrawal from ethanol spares contextual fear conditioning and spatial learning but impairs negative patterning and induces over-responding: Evidence for effect on frontal cortical but not hippocampal function?European Journal of Neuroscience2420521620061688201710.1111/j.1460-9568.2006.04901.xBreeseGROverstreetDHKnappDJNavarroMPrior multiple ethanol withdrawals enhance stress-induced anxiety-like behavior: Inhibition by CRF1- and benzodiazepine-receptor antagonists and a 5-HT1a-receptor agonistNeuropsychopharmacology301662166920051572611410.1038/sj.npp.1300706PMC2864139BrownGJacksonAStephensDNEffects of repeated withdrawal from chronic ethanol on oral self-administration of ethanol on a progressive ratio scheduleBehavioural Pharmacology9149161199810065934ChuKKoobGFColeMDependence-induced increases in ethanol self-administration in mice are blocked by the CRF1 receptor antagonist antalarmin and by CRF1 receptor knockoutPharmacology, Biochemistry & Behavior8681382120071748224810.1016/j.pbb.2007.03.009PMC2170886CiccocioppoRLinDMartin-FardonRWeissFReinstatement of ethanol-seeking behavior by drug cues following single versus multiple ethanol intoxication in the rat: Effects of naltrexonePsychopharmacology (Berlin)16820821520031266419010.1007/s00213-002-1380-zDhaherRFinnDSnellingCHitzemannRLesions of the extended amygdala in C57BL/6J mice do not block the intermittent ethanol vapor-induced increase in ethanol consumptionAlcoholism: Clinical and Experimental Research32197208200810.1111/j.1530-0277.2007.00566.x18162080FinnDASnellingCFretwellAMIncreased drinking during withdrawal from intermittent ethanol exposure is blocked by the CRF receptor antagonist D-Phe-CRF(12-41)Alcoholism: Clinical and Experimental Research3193994920071740306810.1111/j.1530-0277.2007.00379.xFunkCKZorrillaEPLeeMJCorticotropin-releasing factor 1 antagonists selectively reduce ethanol self-administration in ethanol-dependent ratsBiological Psychiatry61788620071687613410.1016/j.biopsych.2006.03.063PMC2741496GriffinWCILopezMFYankeABRepeated cycles of chronic intermittent ethanol exposure in mice increases voluntary ethanol drinking and ethanol concentrations in the nucleus accumbensPsychopharmacology20145698020081879170410.1007/s00213-008-1324-3PMC2590623HeiligMKoobGFA key role for corticotropin-releasing factor in alcohol dependenceTrends in Neuroscience3039940620071762957910.1016/j.tins.2007.06.006PMC2747092HiltunenAJAcute alcohol tolerance in social drinkers: Changes in subjective effects dependent on the alcohol dose and prior alcohol experienceAlcohol143733781997920955310.1016/s0741-8329(96)00186-3JacksonAStephensDNDukaTA low dose alcohol drug discrimination in social drinkers: relationship with subjective effectsPsychopharmacology (Berlin)15741142020011160510110.1007/s002130100817KnappDJOverstreetDHBreeseGRBaclofen blocks expression and sensitization of anxiety-like behavior in an animal model of repeated stress and ethanol withdrawalAlcoholism: Clinical and Experimental Research3158259520071737403710.1111/j.1530-0277.2007.00342.xPMC2864137KoobGFLe MoalMAddiction and the brain antireward systemAnnual Reviews in Psychology59295320081815449810.1146/annurev.psych.59.103006.093548LopezMFBeckerHCEffect of pattern and number of chronic ethanol exposures on subsequent voluntary ethanol intake in C57BL/6J micePsychopharmacology (Berlin)18168869620051600112510.1007/s00213-005-0026-3LopezMFAndersonRIBeckerHCRepeated cycles of chronic intermittent ethanol exposure increase both self-administration and the reinforcing value of ethanol in C57BL/6J miceAlcoholism: Clinical and Experimental Research32163A2008MalcolmRRobertsJSWangWMultiple previous detoxifications are associated with less responsive treatment and heavier drinking during an index outpatient detoxificationAlcohol2215916420001116312310.1016/s0741-8329(00)00114-2MalcolmRMyrickHRobertsJThe effects of carbamazepine and lorazepam on single versus multiple previous alcohol withdrawals in an outpatient randomized trialJournal of General Internal Medicine1734935520021204773110.1046/j.1525-1497.2002.10201.xPMC1495040MalcolmRMyrickLHVeatchLMSelf-reported sleep, sleepiness, and repeated alcohol withdrawals: A randomized, double blind, controlled comparison of lorazepam vs gabapentinJournal of Clinical Sleep Medicine32432200717557449O’DellLERobertsAJSmithRTKoobGFEnhanced alcohol self-administration after intermittent versus continuous alcohol vapor exposureAlcoholism: Clinical and Experimental Research281676168220041554745410.1097/01.alc.0000145781.11923.4eOverstreetDHKnappDJBreeseGRAccentuated decrease in social interaction in rats subjected to repeated ethanol withdrawalsAlcoholism: Clinical and Experimental Research261259126820021219840310.1097/01.ALC.0000023983.10615.D7PMC2865239OverstreetDHKnappDJBreeseGRModulation of multiple ethanol withdrawal-induced anxiety-like behavior by CRF and CRF1 receptorsPharmacology, Biochemistry & Behavior7740541320041475147110.1016/j.pbb.2003.11.010PMC2864717OverstreetDHKnappDJBreeseGRDrug challenges reveal differences in mediation of stress facilitation of voluntary alcohol drinking and withdrawal-induced anxiety in alcohol-preferring P ratsAlcoholism: Clinical and Experimental Research311473148120071762499910.1111/j.1530-0277.2007.00445.xPMC3010749RimondiniRArlindeCSommerWHeiligMLong-lasting increase in voluntary ethanol consumption and transcriptional regulation in the rat brain after intermittent exposure to alcoholFASEB Journal16273520021177293310.1096/fj.01-0593comRimondiniRSommerWHeiligMA temporal threshold for induction of persistent alcohol preference: Behavioral evidence in a rat model of intermittent intoxicationJournal of Studies on Alcohol6444544920031292118510.15288/jsa.2003.64.445RimondiniRSommerWHDall’OlioRHeiligMLong-lasting tolerance to alcohol following a history of dependenceAddiction Biology13263020081785041610.1111/j.1369-1600.2007.00079.xRobertsAJColeMKoobGFIntra-amygdala muscimol decreases operant ethanol self-administration in dependent ratsAlcoholism: Clinical and Experimental Research20128912981996890498410.1111/j.1530-0277.1996.tb01125.xRobertsAJHeyserCJColeMExcessive ethanol drinking following a history of dependence: Animal model of allostasisNeuropsychopharmacology2258159420001078875810.1016/S0893-133X(99)00167-0SchuckitMAKleinJLCorrelations between drinking intensity and reactions to ethanol and diazepam in healthy young menNeuropsychopharmacology415716319912064716SchulteisGLiuJBrain reward deficits accompany withdrawal (hangover) from acute ethanol in ratsAlcohol39212820061693862610.1016/j.alcohol.2006.06.008PMC2266583SommerWHRimondiniRHanssonACUpregulation of voluntary alcohol intake, behavioral sensitivity to stress, and amygdala crhr1 expression following a history of dependenceBiological Psychiatry6313914520081758588610.1016/j.biopsych.2007.01.010StephensDNRipleyTLBorlikovaGRepeated ethanol exposure and withdrawal impairs human fear conditioning and depresses long-term potentiation in rat amygdala and hippocampusBiological Psychiatry5839240020051601897810.1016/j.biopsych.2005.04.025VeatchLMDisruptions in sleep time and sleep architecture in a mouse model of repeated ethanol withdrawalAlcoholism: Clinical and Experimental Research301214122220061679257010.1111/j.1530-0277.2006.00134.xVeatchLMBeckerHCElectrographic and behavioral indices of ethanol withdrawal sensitizationBrain Research94627228220021213793110.1016/s0006-8993(02)02895-0VeatchLMBeckerHCLorazepam and MK-801 effects on behavioral and electro-graphic indices of alcohol withdrawal sensitizationBrain Research10659210620051631388810.1016/j.brainres.2005.10.047WalkerBMKoobGFPharmacological evidence for a motivational role of kappa-opioid systems in ethanol dependenceNeuropsychopharmacology3364365220081747383710.1038/sj.npp.1301438PMC2739278ZhangZMorseACKoobGFSchulteisGDose- and time-dependent expression of anxiety-like behavior in the elevated plus-maze during withdrawal from acute and repeated intermittent ethanol intoxication in ratsAlcoholism: Clinical and Experimental Research311811181920071787778310.1111/j.1530-0277.2007.00483.xPMC2367334

It is well known that alcohol activates the HPA axis, with the magnitude and response profile influenced by a host of variables, including the individual’s specific genetic makeup (i.e., genotype) and sex as well as the alcohol dose ingested (Rivier 2000; Wand 2000). Both clinical and experimental studies have documented profound disturbances in HPA axis function following chronic alcohol exposure and withdrawal. For example, in humans and rodents, chronic alcohol consumption results in a general elevation in blood corticosteroid levels, with a typical flattening of changes in corticosteroid levels that normally is observed throughout the day (Kakihana and Moore 1976; Rasmussen et al. 2000; Tabakoff et al. 1978; Wand and Dobs 1991). At the same time, paradoxically, HPA response to subsequent stress challenge consistently is dampened (i.e., blunted) (Errico et al. 1993; Lee et al. 2000). Whereas the overall heightened HPA axis activation associated with withdrawal usually resolves within a few days (Adinoff et al. 1991; Tabakoff et al. 1978; Willenbring et al. 1984), the blunted responsiveness of the HPA axis to subsequent challenges appears to persist for a protracted period of time (Adinoff et al. 1990; Costa et al. 1996; Lovallo et al. 2000). In some cases, this may be accompanied by reduced basal levels of circulating corticosteroids (Marchesi et al. 1997; Rasmussen et al. 2000; Zorrilla et al. 2001).

In addition to these HPA axis–related effects, alcohol alters CRF activity independent of the HPA axis (Heilig and Koob 2007; Koob and Le Moal 2001). CRF is a 41–amino acid neuropeptide that is widely distributed throughout the mammalian brain and plays a critical role not only in regulating HPA axis activity but also in orchestrating other behavioral and physiological responses to stress. To exert these effects, CRF interacts with two types of receptors called CRF_1_ and CRF_2_ receptors that are located in the membrane surrounding the target cells on which CRF acts. Outside of the hypothalamus, CRF and its receptors are found in an extensive network of interconnected neural structures that are intimately associated with the brain’s reward and stress pathways, such as the amygdala, bed nucleus of stria terminalis (BNST), and prefrontal cortex. Following chronic alcohol exposure, increased CRF release, along with an increase in the number (i.e., upregulation) of CRF_1_ receptors, can be observed, especially in these brain areas. These variations represent an important neuroadaptive change (Heilig and Koob 2007; Koob and Le Moal 2001) that is thought to be key in the emergence of withdrawal-related anxiety and dysphoria, both of which likely are intimately tied to alcohol drinking and relapse (Becker 1999; Koob 2003). The contribution of CRF to withdrawal-related anxiety is supported by findings that agents which interfere with the normal actions of CRF (i.e., CRF antagonists) can reduce the anxiety if they are administered into the blood (i.e., systemically) (Breese et al. 2005; Sommer et al. 2008) or directly into the CNS—that is, either into the fluid-filled spaces of the brain (i.e., brain ventricles) (Baldwin et al. 1991; Valdez et al. 2003) or into the central nucleus of the amygdala (Rassnick et al. 1993). This effect appears to be mediated by CRF_1_ receptors because CRF antagonists that selectively block CRF_1_ receptors result in anxiety reduction (Overstreet et al. 2004). Conversely, activation of CRF_2_ receptors may attenuate withdrawal-related anxiety (Valdez et al. 2004). Thus, chronic alcohol exposure and withdrawal experiences can be viewed as potent stressors that disrupt the functional integrity of the HPA axis and also act on the extrahypothalamic CRF systems. This perturbation in the brain and hormonal (i.e., neuroendocrine) stress axes may have significant implications for motivation for alcohol self-administration behavior.

Although the circumstances and manner in which stress influences drinking behavior are complex and not fully understood, it generally is acknowledged that stressful life events prominently influence alcohol drinking and, in particular, may trigger relapse (Brady and Sonne 1999; Sillaber and Henniger 2004; Sinha 2001; Weiss 2005). Activation of the HPA axis and CRF-related brain stress circuitry resulting from alcohol dependence likely contributes to amplified motivation to drink. For example, animal studies have indicated that elevation of corticosteroid hormone levels may enhance the propensity to drink through an interaction with the brain’s main reward circuitry (i.e., mesocorticolimbic dopamine system) (Fahlke et al. 1996; Piazza and Le Moal 1997). A CRF antagonist that acts on both the CRF_1_ and CRF_2_ receptors (i.e., a nonselective peptide CRF antagonist) called D-Phe-CRF_12–42_ reduced excessive drinking in dependent animals when administered into the brain ventricles (Finn et al. 2007; Valdez et al. 2002) or the central nucleus of the amygdala (Funk et al. 2006). Similarly, systemic administration of antagonists that selectively act at the CRF_1_ receptor also reduced upregulated drinking in dependent mice (Chu et al. 2007) and rats (Funk et al. 2007; Gehlert et al. 2007).

Different stressors likewise robustly reinstated extinguished alcohol-reinforced responding in different operant reinstatement models of relapse (Funk et al. 2005; Gehlert et al. 2007; Le et al. 2000, 2005; Liu and Weiss 2002*b*). This effect appears to involve CRF activity because CRF antagonists block stress-induced reinstatement of alcohol-seeking behavior (Gehlert et al. 2007; Le et al. 2000; Liu and Weiss 2002*b*). Moreover, extrahypothalamic CRF activity appears to contribute to this effect because surgical removal of the adrenal gland (i.e., adrenalectomy), which renders the HPA axis nonfunctional, did not affect the stress-induced reinstatement of alcohol-seeking behavior (Le et al. 2000).^4^ Finally, direct infusion of CRF antagonists into a brain region called the median raphe nucleus^5^ blocked stress-induced alcohol-seeking behavior, possibly by interacting with CRF_1_ receptors (Le et al. 2002; Marinelli et al. 2007*a*).

Taken together, a substantial body of evidence suggests that changes in CRF function within the brain and neuroendocrine systems may influence motivation to resume alcohol self-administration either directly and/or by mediating withdrawal-related anxiety and stress/dysphoria responses.

## Treatment Implications

Relapse represents a major challenge to treatment efforts for people suffering from alcohol dependence. To date, no therapeutic interventions can fully prevent relapse, sustain abstinence, or temper the amount of drinking when a “slip” occurs. For some people, loss of control over alcohol consumption can lead to alcohol dependence, rendering them more susceptible to relapse as well as more vulnerable to engaging in drinking behavior that often spirals out of control. Many of these people make numerous attempts to curtail their alcohol use, only to find themselves reverting to patterns of excessive consumption.

Significant advancements have been made in understanding the neurobiological underpinnings and environmental factors that influence motivation to drink as well as the consequences of excessive alcohol use. Given the diverse and widespread neuroadaptive changes that are set in motion as a consequence of chronic alcohol exposure and withdrawal, it perhaps is not surprising that no single pharmacological agent has proven to be fully successful in the treatment of alcoholism. The challenge of choosing the most appropriate agent for the treatment of alcoholism is compounded by the complexity and heterogeneity of this relapsing disease as well as by the host of other variables (e.g., genotype, coexisting disorders, treatment regimens, and compliance) that must be considered in the context of treatment interventions (e.g., McLellan et al. 2000). Further, the efficacy of treatment may depend on temporal factors, such as the stage of addiction (e.g., whether the patient seeks treatment or not) as well as drinking pattern (e.g., binge-like intake) (Anton et al. 2004), especially when both amount and frequency of alcohol consumption is assessed to determine drinking behavior/phenotype (Feunekes et al. 1999).

Nevertheless, numerous pharmacotherapies have been employed to treat alcoholism, guided principally by advancing knowledge about alcohol’s interactions with various components of the brain’s reward and stress pathways (Heilig and Egli 2006; Litten et al. 2005; Spanagel and Kiefer 2008). To date, two medications targeting these brain systems—naltrexone (Revia^®^) and acamprosate (Campral^®^)—have been approved by the Food and Drug Administration (FDA) for treatment of alcoholism.^6^ The efficacy of naltrexone and acamprosate in treating alcohol dependence and relapse is based on numerous clinical studies, although support is not universal (Anton et al. 2006; Heilig and Egli 2006; Mann et al. 2008; Spanagel and Kiefer 2008). Naltrexone operates as an antagonist of certain receptors (principally μ and δ receptors) for brain-signaling molecules (i.e., neurotransmitters) called endogenous opiates that are involved in reward systems, whereas acamprosate is thought to modulate signal transmission involving another neurotransmitter called glutamate. It has been postulated that naltrexone may blunt the rewarding effects of alcohol, whereas acamprosate may attenuate adaptive changes during abstinence that favor relapse (Heilig and Egli 2006; Litten et al. 2005).

As previously indicated, a variety of animal models have been used to study the ability of these and other medications to reduce alcohol consumption as well as prevent and/or retard relapse. Of particular interest are studies demonstrating that animals with a history of dependence exhibit greater sensitivity to some medications that impact alcohol relapse–like behavior compared with animals without such a history (Ciccocioppo et al. 2003; Funk et al. 2007; Gehlert et al. 2007; Liu and Weiss 2002*a*, *b*). These findings raise the promising prospect that therapeutics may be developed which specifically target excessive uncontrolled alcohol drinking without producing nonspecific effects (i.e., without reducing certain behaviors in dependent as well as nondependent subjects). Further advances in understanding the neurobiological factors that bear on the complex problem of relapse will no doubt continue to enlighten and facilitate discovery of new and more effective treatment strategies for controlling excessive drinking associated with alcohol dependence.

## Summary

A complex interplay among numerous biological and environmental factors governs the motivational aspects of alcohol-seeking and drinking behavior throughout the addiction process. Chronic excessive alcohol consumption can lead to the development of dependence. When drinking is terminated, a characteristic withdrawal syndrome ensues that includes potentially life-threatening physical symptoms as well as a constellation of symptoms that contribute to psychological distress, anxiety, and negative affect. Many withdrawal symptoms associated with this negative emotional state persist for a long period of time and constitute a powerful motivational force promoting the perpetuation of alcohol use/abuse as well as enhancing vulnerability to relapse. Both clinical studies and basic research studies using animal models have demonstrated that alcohol-related (conditioned) cues and contexts as well as stressful stimuli and events can trigger relapse. Moreover, a history of dependence appears to amplify responsiveness to such relapse-provoking stimuli and events.

Alcohol dependence is thought to represent a persistent dysfunctional (i.e., allostatic) state in which the organism is ill-equipped to exert appropriate behavioral control over alcohol drinking. Functional changes in brain and neuroendocrine stress and reward systems as a result of chronic alcohol exposure and withdrawal play a key role not only in altering the rewarding effects of alcohol, but also in mediating the expression of various withdrawal symptoms that, in turn, impact motivation to resume drinking. Although currently few treatments are available for tackling this significant health problem and providing relief for those suffering from the disease, there is hope. As new and exciting discoveries in neuroscience, genetics, neuroimaging, and biological psychiatry/psychology continue to advance understanding of the complexities of alcohol dependence, new insights will emerge that point to novel targets for the next generation of therapeutics, which hopefully will be more effective in preventing relapse and/or tempering alcohol intake in people attempting to control their drinking problems.

## Footnotes

1 To be diagnosed with alcohol dependence according to the *Diagnostic and Statistical Manual of Mental Disorders, 4th Edition* (DSM–IV) (American Psychiatric Association 1994), an individual must meet at least four of the following criteria: drinking more alcohol than intended, unsuccessful efforts to reduce alcohol drinking, giving up other activities in favor of drinking alcohol, spending a great deal of time obtaining and drinking alcohol, continuing to drink alcohol in spite of adverse physical and social effects, and the development of alcohol tolerance.

2 The autonomic nervous system is that division of the nervous system which regulates the functions of the internal organs and controls essential and involuntary bodily functions, such as respiration, blood pressure and heart rate, or digestion.

3 In operant procedures, animals must first perform certain response (e.g., press a lever) before they receive a stimulus (e.g., a small amount of alcohol). By modifying the required response (e.g., increasing the number of lever presses required before the alcohol is delivered) researchers can determine the motivational value of the stimulus for the animal.
